# A pilot prospective study of sleep patterns and DNA methylation-characterized epigenetic aging in young adults

**DOI:** 10.1186/s13104-019-4633-1

**Published:** 2019-09-16

**Authors:** Mary A. Carskadon, Kenneth R. Chappell, David H. Barker, Anne C. Hart, Kayla Dwyer, Caroline Gredvig-Ardito, Caitlyn Starr, John E. McGeary

**Affiliations:** 1 EP Bradley Hospital Sleep Research Laboratory, 300 Duncan Drive, Providence, RI 02906 USA; 2 Department of Psychiatry and Human Behavior, Alpert Medical School of Brown University, Box G-A1, Providence, RI 02912 USA; 3 Bradley Hasbro Children’s Research Center, CoroWest, 1 Hoppin Street, Suite 204, Providence, RI 20903 USA; 4 Department of Neuroscience and Robert J. & Nancy D. Carney Institute for Brain Science, Brown University, 185 Meeting Street, Providence, RI 02912 USA; 5 Providence Veterans Affairs Medical Center, 830 Chalkstone Avenue, Providence, RI 02098 USA

**Keywords:** Sleep regularity, Sleep duration, Epigenetic aging, Young adult

## Abstract

**Objective:**

Molecular markers in DNA methylation at a subset of CpG sites are affected by the environment and contribute to biological (epigenetic) age. We hypothesized that shorter sleep duration and possibly irregular sleep would be associated with accelerated epigenetic aging. We examined epigenetic vs. chronological age in 12 young women selected as shorter or longer sleepers studied prospectively across the first 9 weeks of college using a daily online sleep log. Genomic DNA was isolated from two blood samples spanning the interval, and DNA methylation levels were determined and used to measure epigenetic age.

**Results:**

Epigenetic vs. chronological age differences averaged 2.07 at Time 1 and 1.21 at Time 2. Sleep duration was computed as average daily total sleep time and sleep regularity was indexed using the Sleep Regularity Index. Participants with longer and more regular sleep showed reduced age difference: mean = − 2.48 [95% CI − 6.11; 1.15]; those with shorter and more irregular sleep showed an increased age difference: 3.03 [0.02; 6.03]; and those with either shorter or more irregular sleep averaged no significant change: − 0.49 [− 3.55; 2.56]. These pilot data suggest that short and irregular sleep, even in a young healthy sample, may be associated with accelerated epigenetic aging.

## Introduction

The study of aging characterizes developmental changes over time and often focuses on the relationship of aging to mortality and morbidity. A subfield of aging research known as geroscience or biogerontology has identified molecular markers that reliably track with normal chronological aging. These molecular markers include changes in DNA methylation at a subset of CpG sites, which can alter expression of specific genes. While DNA methylation changes seen in the genome can reflect normal cell differentiation, a subset of CpG sites have methylation alterations due to environmental exposures. In particular, Horvath [1] and others identified a subset of these malleable methylation changes that track with organismic aging. Though these markers tend to correspond generally with chronological age, they are influenced by environment, contributing to discrepancies between biological age (i.e., epigenetic age) and chronological age.

Many factors have been identified that are associated with an epigenetic age that exceeds chronological age (accelerated aging). These factors include disease states such as cancer [2], diabetes [3], and insomnia [4], as well as poor health indices such as triglycerides and systolic blood pressure [5], along with lifestyle factors that include inactivity, smoking, and diet [5]. Moreover, Chen et al. [6], found the Horvath index of epigenetic age predicted all-cause mortality after accounting for chronological age. Taken together, these findings suggest that factors associated with poor health are also associated with accelerated aging which in turn predicts mortality.

Less work has been done in identifying factors associated with reduced aging (epigenetic age < chronological age). Cross sectional studies suggest that regular meditation may be associated with slower epigenetic aging [7]. Additionally, exercise interventions associated with reduced DNA methylation at some CpG sites [8] hint that age-related CpG sites might be impacted through behavioral interventions. Some cellular models agree that molecular manipulations may lead to slower epigenetic aging [1, 9, 10]. More work is needed to (1) identify reliable factors that slow or accelerate epigenetic age, (2) characterize the magnitude of effect of these factors, and (3) determine the timeframe of change in epigenetic aging.

## Main text

In a previous study, using a prospective design, we identified differentially methylated genes in young adults associated with shorter or longer sleep duration during the first semester of college [11]. In the present study, we interrogate methylomic data of twelve women from this sample to determine epigenetic ages before and after a 9-week span during which participants tracked their sleep, hence providing a prospective analysis of how sleep patterns relate to epigenetic aging. Our hypothesis is that those with shorter sleep duration would show accelerated epigenetic aging across the 9 weeks and those with longer sleep duration would show slower epigenetic aging. Furthermore, we hypothesize that the regularity of sleep across this interval might similarly affect epigenetic aging, with more irregularity accelerating aging.

### Methods

The parent study recruited first-year Brown University students to track their sleep across the first 9 weeks of the first semester with an on-line daily sleep diary (bedtime, rising time, napping, etc.) starting the first day of class and ending before the Thanksgiving holiday break. The study took place with new cohorts recruited across 3 years (2012–2014). Each participant’s initial visit included review of procedures, signing consent, and obtaining a blood sample. Within a week of ending the diary completion, each participant provided a second blood sample. Participants provided written consent. All procedures were approved by the Lifespan Institutional Review Board for Protection of Human Subjects.

A total of 503 participants completed the study (i.e., provided > 75% of daily diaries, completed the final online questionnaire, and gave both blood samples). The participants in this pilot methylomic study included 12 females who self-identified as white, four of whom also endorsed Hispanic ethnicity. Chronological ages of these participants at the time of the first blood draw ranged from 18.18 to 19.84 years (Mean = 18.72 [SD = 0.44]). Six women were in the upper quintile (Longer Sleepers) and 6 in the lower quintile (Shorter Sleepers) of average total sleep time (TST) of the entire sample of 503 participants. Selection was performed before running methylomic analysis.

Although not used in participant selection, we also examined sleep regularity from the sleep diary data using the Sleep Regularity Index (SRI) [12]. A score of 100 on this scale indicates perfect alignment in the timing of all sleep episodes; lower scores indicate greater sleep irregularity. Because the diaries provided information about the number and duration, but not timing of naps and night awakenings, the SRI was modified so that sleep and wake index values used in the SRI calculation were multiplied by the proportion of the sleep or wake period spent in the opposing state. Missing diary days were coded as missing and did not contribute to the calculation of the SRI.

Genomic DNA was isolated (DNEasy Blood and Tissue Kit, Qiagen) from the whole blood samples. DNA methylation levels were determined for each group using the Infinium HumanMethylation450 platform (Illumina, San Diego). To determine Horvath DNA methylation ages, IDAT files for each time point from the 12 subjects were loaded into R v3.4.3 via minfi v1.20.2 [13–15]. Beta values were retrieved after quantile normalization and estimated sample blood cell counts were obtained [15, 16]. Flow-sorted beta values from FlowSorted.Blood.450k v1.12.0 were retrieved after quantile normalization [17]. Sample beta values underwent BMIQ normalization and were then corrected for cell type using flow-sorted and estimated cell count data [1, 18]. Corrected beta values were used to estimate epigenetic age with the age transformation function. Non-corrected, quantile-normalized beta values were uploaded to the online age calculator for comparison [19]. Chronologic ages were subtracted from epigenetic ages to provide age-difference for analysis (i.e., those with accelerated age had higher epigenetic ages than chronologic ages and thus had a positive value). Minimum Information About a Microarray Experiment (MIAME) documentation can be found at https://www.ncbi.nlm.nih.gov/geo/query/acc.cgi?acc=GSE80559.

### Results

Results focus on the outcome of age-difference (epigenetic age—chronological age). To examine the relation between sleep and age-difference, we categorized participants into groups based on TST selection (Longer Sleepers mean TST = 8.04 [SD = 0.24] vs. Shorter Sleepers = 5.95 [0.62]) and median split for SRI (median = 76.44; More Regulated SRI = 81.3 [3.02] vs. Less Regulated = 66.2 [7.92]). Crossing these splits resulted in three equally sized groups: Good Sleep (Longer Sleepers and More Regulated; TST = 7.97 [0.13]; SRI = 80.62 [3.07]), Mixed Sleep (either Longer Sleeper or More Regulated; TST = 6.93 [1.60]; SRI = 74.78 [9.55]), and Poor Sleep (Shorter Sleeper and Less Regulated; TST = 6.08 [0.38]; SRI = 65.84 [9.88]). Analyses of variance were used to examine the association between sleep groups and age-difference at Time 1. Analyses of covariance were used to examine change in epigenetic aging across the 9-weeks, with the outcome being the change in age-difference from Time 1 to Time 2 with Time 1 being included as a covariate. All analyses were performed using R v3.5.1.

**Fig. 1:**
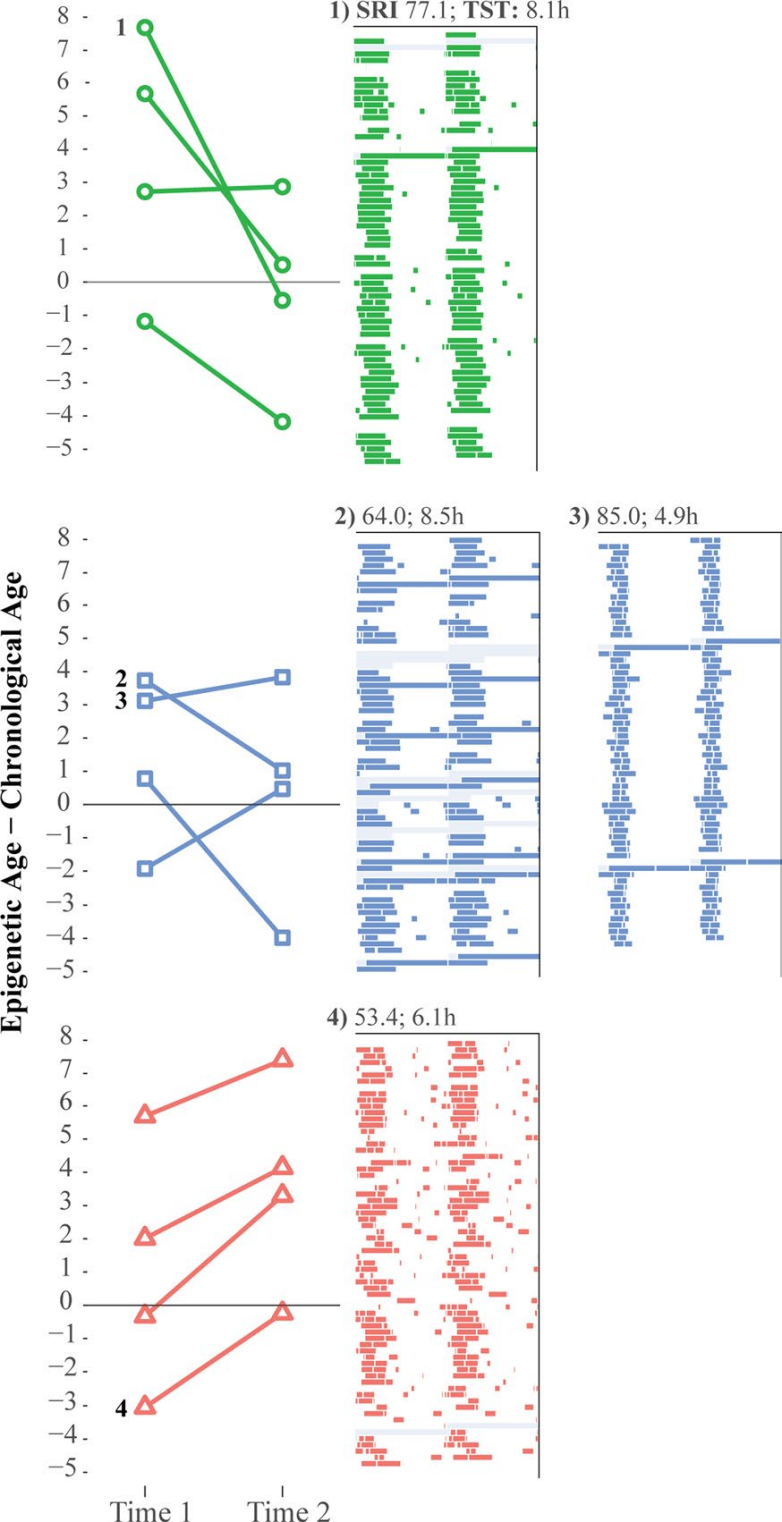
Age-differences across 9-weeks by sleep category. Individual participant values of age-differences for each time point are depicted by sleep group. Top panel (Good Sleep) are those above the median on *both* total sleep time (TST) and the sleep regularity index (SRI). Middle panel (Mixed Sleep) are those above the median on *either* TST or SRI. Bottom panel (Poor Sleep) are those below the median on *both* TST and SRI. Double plotted raster plots are provided for exemplar sleep patterns in each sleep group, with numbers linking aging data to the raster plots. Gray indicates missing diary data. Because timing data was not available for naps and night awakenings, timing was randomly allocated during the respective sleep or wake period in raster plots

Demographics and Sleep data for each participant are listed in Table 1. Age related data are listed in Table 2. Chronological age and epigenetic age at the time of the first blood sample (Time 1) were not correlated (*r *= − 0.05). Epigenetic ages at Time 1 ranged from 15.79 to 26.29 years (mean = 20.79 [SD = 3.28]); epigenetic ages computed from the Time 2 samples ranged from 16.03 to 25.93 years (20.12 [3.16]). Mean age-difference for Time 1 was 2.07 [3.33] years and 1.21 [3.35] at Time 2. Age-difference values for each participant at each assessment by sleep category are depicted in Fig. 1 along with exemplar sleep raster plots that illustrate both sleep duration and sleep regularity. There were no differences in age-difference at Time 1 for TST (Shorter Sleepers vs. Longer Sleepers; *F*(1,10) = 0.52, *p *= 0.49), SRI (More Regulated vs. Less Regulated; *F*(1,10) = 1.23, *p *= 0.29), or for the three sleep groups defined by TST and SRI (*F*(2,9) = 0.70, *p *= 0.52). Patterns of epigenetic aging across the 9-week interval were consistent with hypotheses: those in the Good Sleep group generally showed decreases in age-difference across time, the Mixed Sleep group showed inconsistent patterns of change, and those in the Poor Sleep group showed increases in age-difference. The observed differences in these group patterns were significant (*F*(2,8) = 4.97, *p *= 0.03; Marginal Mean Estimate [95% CI]; Good Sleep = − 2.48 [− 6.11; 1.15]; Mixed Sleep = − 0.49 [− 3.55; 2.56]; Poor Sleep = 3.03 [0.02; 6.03]). Examining TST seperately from SRI indicated that Shorter Sleepers showed an increase in epigenetic age of 3.03 [− 1.02; 7.07] compared to Longer Sleepers (*F*(1,9) = 4.72, *p *= 0.06) and Less Regulated sleepers showed an increase in epigenetic age of 4.13 [0.48; 7.78] compared to More Regulated sleepers (*F*(1,9) = 10.99, *p *= 0.01). As a sensitivity check, we reran all analyses using GrimAge [20], an alternative method for scoring epigenetic age. Results were similar and did not alter our conclusions. Chronologic age and the epigenetic ages defined using the Horvath scoring and GrimAge scoring for samples at each time point are listed in Table 2.

**Table 1:** Demographic and sleep data

Patient	Hispanic	SRI	TST	Waketime	Bedtime
1	1	62.76	5.96	8.04	01.85
2	0	85.04	4.87	8.14	03.06
3	1	77.11	8.11	9.48	24.77
4	1	84.53	7.98	9.91	01.79
5	0	63.93	8.47	9.4	24.76
6	0	80.09	6.48	7.84	01.08
7	0	80.91	7.79	8.99	24.95
8	1	53.43	6.11	7.94	01.63
9	0	75.77	6.58	8.36	01.45
10	0	70.04	7.88	8.97	24.6
11	0	71.4	5.68	9.13	03.05
12	0	79.91	7.99	9.84	01.54

**Table 2:** Aging data

Chronological age		Epigenetic age (Horvath)		Epigenetic age (GrimAge)	
Time 1	Time 2	Time 1	Time 2	Time 1	Time 2
18.87	19.06	20.89	23.20	20.46	22.28
18.67	18.87	21.79	22.70	21.35	22.24
18.62	18.81	26.29	18.26	26.51	17.99
18.61	18.81	17.43	14.61	17.41	15.43
18.26	18.46	21.99	19.48	22.54	19.70
19.84	20.04	20.62	16.03	20.84	16.81
19.03	19.23	24.71	19.75	25.22	20.11
18.86	19.05	15.79	18.81	16.41	19.56
18.85	19.05	18.52	22.35	18.69	22.05
18.44	18.64	16.51	19.11	16.79	19.27
18.35	18.55	24.04	25.93	23.55	25.50
18.18	18.37	20.89	21.24	21.14	21.57

### Discussion

By using a prospective design, our pilot data provide unique, albeit limited, evidence connecting sleep patterns over a relatively short timeframe to molecular aging indices. Change in epigenetic age over 9 weeks was associated with distinct differences in daily self-reported sleep. Poorer sleep was associated with marked acceleration of epigenetic aging in four of four participants; whereas better sleep, was associated with decelerated epigenetic aging in three of four participants.

We note that our participants were similar in many respects: all were first-year university students, young (chronologic age of 18–19 years), and women who self-identified as white (four reporting Hispanic ethnicity). They were selected from a much larger parent sample group to have marked differences in sleep duration across the 9-week interval. These pilot data add to a growing literature highlighting the adverse health effects of short, disrupted, or disordered sleep by indicating that short and irregular sleep, even in a young healthy sample, appears to be related to accelerated epigenetic aging. Epigenetic aging, in turn, has been linked with important health markers including all-cause mortality [6]. More work is needed to confirm these findings in larger samples, to identify the mechanisms through which sleep behavior influences epigenetic aging, and to determine whether behavioral sleep interventions can slow epigenetic aging.

## Limitations

The primary limitation of these data is the small sample, which precluding analysis of patient characteristics (e.g., stress, physical health, previous sleep history) that may relate to both sleep and epigenetic aging. We were also not able to account for technical variables (e.g., batch effects, timing of blood draw) that may influence the accuracy of the epigenetic results. The size of the sample was dictated by the cost of methylomic analyses and limited budget for the pilot project. Although the sample size is limited, this pilot study has a number of strengths including a prospective design and use of daily sleep diaries across 9-weeks. To our knowledge, there are no other prospective data in which to examine the association between sleep and epigenetic aging.

## Data Availability

All data used in analyses are presented in Table 1. Minimum Information About a Microarray Experiment (MIAME) documentation can be found at https://www.ncbi.nlm.nih.gov/geo/query/acc.cgi?acc=GSE80559.
